# Supplementary material for: Ultrabroadband on-chip photonics for full-spectrum wireless communications
Source: Nature. 2025 Aug 27;645(8079):80–7. doi: 10.1038/s41586-025-09451-8 (PMC12408381; doi:10.1038/s41586-025-09451-8)
Supplement: Supplementary file 1 — Supplementary Information [file 41586_2025_9451_MOESM1_ESM.pdf]

nature portfolio

https://doi.org/10.1038/s41586-025-09451-8

Supplementary information

Ultrabroadband on-chip photonics for full-spectrum wireless communications

In the format provided by the authors and unedited

Supplementary Information for

Ultrabroadband on-chip photonics for full-spectrum wireless communications

Zihan Tao[1,†], Haoyu Wang[1,†], Hanke Feng[2,†], Yijun Guo[1,†], Bitao Shen[1,†], Dan Sun[3], Yuansheng Tao[2], Changhao Han[4], Yandong He[5], John E. Bowers[4], Haowen Shu[1,6,*], Cheng Wang[2,*] & Xingjun Wang[1,3,6,*]

[1]State Key Laboratory of Advanced Optical Communications System and Networks, School of Electronics, Peking University, Beijing, China.

[2]Department of Electrical Engineering State & Key Laboratory of Terahertz and Millimeter Waves, City University of Hong Kong, Kowloon, China.

[3]Peking University Yangtze Delta Institute of Optoelectronics, Nantong, China.

[4]Department of Electrical and Computer Engineering, University of California Santa Barbara, Santa Barbara, CA, USA.

[5]School of Integrated Circuits, Peking University, Beijing, China.

[6]Frontiers Science Center for Nano-optoelectronics, Peking University, Beijing, China.

[†]These authors contributed equally to this work.

Corresponding authors: *haowenshu@pku.edu.cn, *cwang257@cityu.edu.hk, *xjwang@pku.edu.cn

Supplementary note I: Fabrication detail of TFLN chip

Devices are fabricated from a commercially available x-cut LNOI wafer (NANOLN), with a 500 nm LN thin film, a 4.7 µm buried SiO_2 layer, and a 500-µm silicon substrate. SiO_2 is first deposited on the surface of a 4-inch LNOI wafer as etching hard mask using plasma-enhanced chemical vapor deposition (PECVD). Optical waveguides, MZIs and microring resonators are then patterned on the entire wafer using an ASML UV Stepper lithography system (NFF, HKUST) die by die (1.5 cm $\times$ 1.5 cm) with a resolution of 500 nm. Next, the exposed resist patterns are transferred first to the SiO_2 layer using a standard fluorine-based dry etching process, and then to the LN device layer using an optimized Ar^+-based inductively-coupled plasma (ICP) reactive-ion etching process. The LN etch depth is around 250 nm, leaving a 250-nm-thick slab. After removal of the residual SiO_2 mask and redeposition, an annealing process is carried out. Afterward, a second, third, and fourth lithography and lift-off process is used to fabricate the microwave electrodes, heater, and wires/pads, respectively. The advanced slotted-electrodes are employed to increase the E-O bandwidth with 5 µm slot length and 2 µm slot width[1]. The positive and negative electrodes are spaced by a gap of 5 µm to ensure strong E-O coupling while minimizing metal-induced optical losses. Fig.S1 is the fabricated waveguide (i) and electrodes (ii).

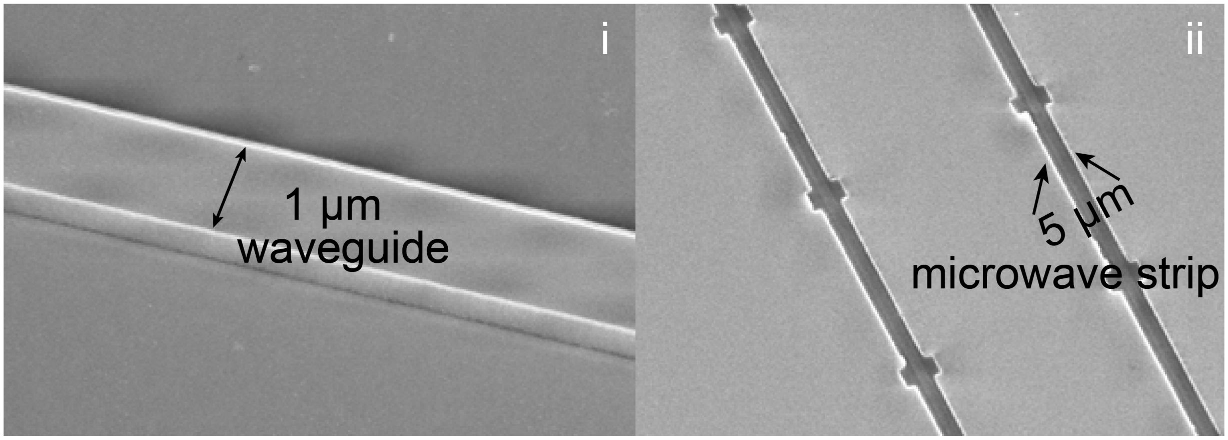

Fig. S1. Scanning electron microscope images of the fabricated waveguide (i) and microwave electrode (ii).

Supplementary note II: Performance Characterization Details of TFLN Chips

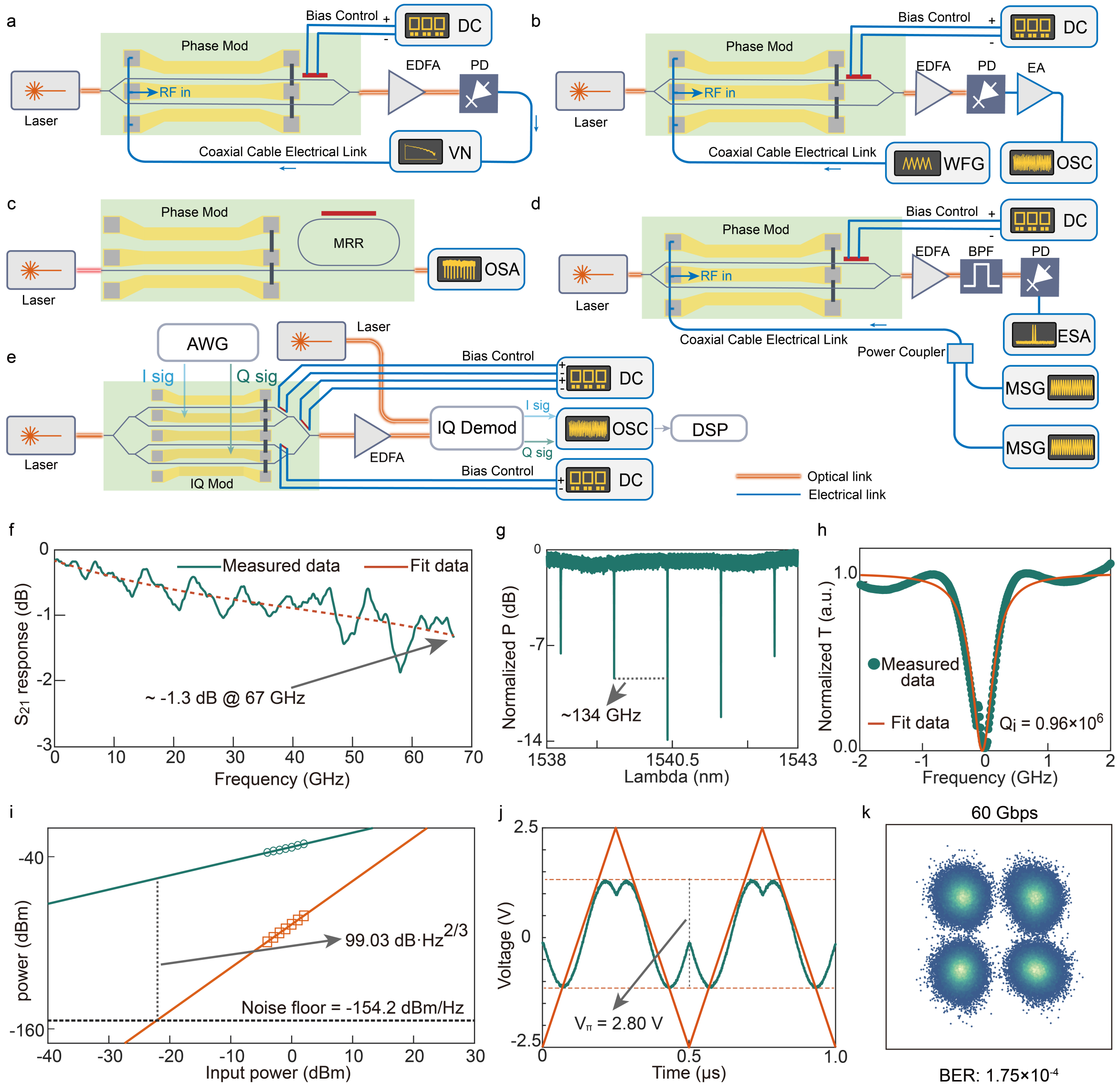

Fig. S2. **Experimental setup and results for characterizing key component performance. a,** Setup for E-O bandwidth measurement of TFLN-MZM. **b,** Setup for V_π measurement of TFLN-MZM. **c,** Setup for FSR and Q measurement of microring. **d,** Setup for linearity measurement of TFLN-MZM. **e,** Setup of communication test using on-chip IQ modulator. **f,** Bandwidth measurement result of the E-O modulator. **g,** FSR measurement result of the on-chip microring resonator. **h,** Quality factor measurement result of the on-chip microring resonator. **i,** Linearity measurement result of the E-O modulator. **j,** $V\pi$ measurement result of the E-O modulator. **k,** Fiber-optic communication performance of the on-chip IQ modulator. DC: direct current; VNA: vector network analyzer; WFG: waveform generator; OSC: oscilloscope; ESA: electronic spectrum analyzer; MSG: microwave signal generator.

E-O bandwidth measurement: To determine the 3-dB bandwidth of the fabricated TFLN-based modulator, we employed a vector network analyzer (VNA, Keysight N5227B) to supply an

RF signal to the modulator's input through a high-frequency probe, while a 50 Ω matching resistor was connected at the output to ensure impedance matching. The optical signal output from the modulator is amplified by an EDFA and then enters a high-speed photodetector, converting the optical signal into an electrical signal, which is fed back into the VNA for S-parameter analysis. Experimental setup is shown in Fig.S2a. Through the mentioned procedure, the E-O bandwidth is obtained. The measured 1.3 dB bandwidth reaches 67 GHz, as shown in Fig.S2f.

FSR and Q factor measurement: The free spectral range (FSR) and Q factor measurement is performed by scanning a tunable laser (Santec TSL 570) across a resonance from the blue side to the red side. To avoid the distortion of line shape caused by thermal nonlinearity, the on-chip power is reduced to around -25 dBm. We use a optical spectrum analyzer (OSA) to acquire the spectrum transmitted through the on-chip microring, where the OSA is implemented with a multi-channel optical power meter (Santec MPM 210) and a computer that supports synchronization with the laser. Experimental setup is shown in Fig.S2c. The on-chip microring resonator is found to have a FSR of 134 GHz through measuring the spacing between resonance peaks in the spectral data, as shown in Fig.S2g. Then the spectral data is subsequently loaded into MATLAB for Lorentzian fitting according to the following formula:

$$y = y_0 + \frac{2A}{\pi} \frac{\Delta f}{4\left(f - f_c\right)^2 + \Delta f^2} \tag{1}$$

where A, Δf, f_c, and y_0 represent the amplitude, full width at half maximum, center frequency, and amplitude offset of the Lorentzian curve, respectively. Analysis of the fitted data reveals that the resonance exhibits a mean full width at half maximum (FWHM) of 406.8 MHz under critical coupling conditions at around 1540.5 nm, corresponding to a loaded Q-factor (Q_L) of 0.48×10^6 and an intrinsic Q-factor (Q_i) of 0.96×10^6, as shown in Fig.S2h.

Spurious-free dynamic range (SFDR) measurement: SFDR is obtained using a two-tone test method. First, two microwave signals with closely frequency are generated by two microwave signal sources (Keysight E857D; Anristu MG3695A). These are combined into one signal via a power combiner, which is then applied to the modulator. A spectrum analyzer(Keysight N9010B) is used to capture the spectrum of the detector's demodulated output signal and to analyze the power values of the fundamental component and the nonlinear distortion third-order intermodulation distortions (IMD3) frequency component. Experimental setup is shown in Fig.S2d. The power values of the fundamental and nonlinear IMD3 frequency components are measured at various input power levels, followed by linear fitting of the measured data. The overall noise floor of the

link was measured at -154.2 dBm/Hz, and the SFDR is approximately 99.03 dB·Hz$^{2/3}$, as shown in Fig.S2i.

V_π **measurement:** For the measurement of V_π, we use a waveform generator that produces a triangular wave with a Vpp of 5 V and a frequency of 200 kHz, which is loaded onto the modulator via a high-frequency probe. Since the IQ modulator consists of two parallel MZMs, we measured the V_π of adjacent MZM modulators on the same chip to represent the V_π characteristics of both the IQ modulator and the entire chip. The modulator's output signal is sent to the oscilloscope and displayed in sync with the signal from the signal generator. The experimental setup is shown in Fig.S2b. The triangular wave voltage required for the peak-to-peak value of the output curve is the half-wave voltage of the modulator. The measured half-wave voltage is 2.8V, as shown in Fig.S2j.

Details of transmission using on-chip IQ modulator: The IQ modulator consists of two TFLN MZM intensity modulators, with one serving as the in-phase component and the other as the quadrature component. To characterize the communication performance of the on-chip IQ modulator, we conducted fiber-optic communication experiments. A thermal phase shifter creates a 90° phase difference between the two paths. To achieve IQ modulation, we adjust the thermal tuning of the two intensity modulators to operate at their null points. For signal transmission, the two output signals from the AWG are applied to the I and Q arms of the modulator. The modulated optical signal is amplified by an EDFA, then enters the coherent receiver. It is captured by an oscilloscope and processed offline to achieve communication functionality. Experimental setup is shown in Fig.S2e. By precisely controlling the bias points of the IQ modulator, we achieved a maximum transmission rate of 60 Gbps with a BER of around 1.75×10^{-4}, as shown in Fig.S2k. The result can be further enhanced by correcting the IQ imbalance using a bias point controller with an automatic feedback loop.

Supplementary note III: The overall link noise figure analyzes

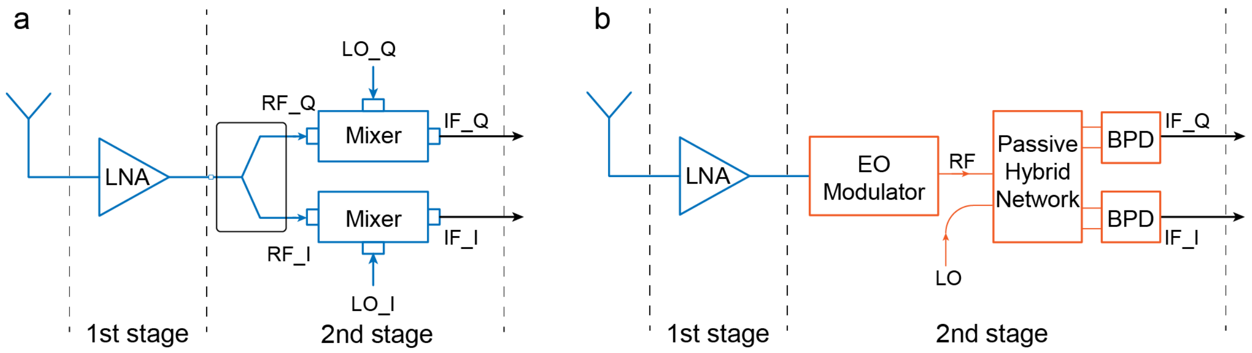

Fig. S3. Different cascaded system of electronic system (**a**) and photonic-assisted system (**b**)

The overall noise figure (NF) of a multi-stage RF system can be calculated using the Friis equation in linear form:

$$F = F_1 + \frac{F_2 - 1}{G_1} + \frac{F_3 - 1}{G_1 G_2} + \cdots + \frac{F_N - 1}{G_1 \cdots G_{N-1}} \tag{2}$$

where G_i, F_i represent the gain and noise figure of the ith stage, respectively. Since the first-stage gain typically dominates, a low-noise amplifier (LNA) is employed as the first stage. In this work, a commercial wideband LNA with a gain of 35 dB, NF of 4 dB, and a 3-dB bandwidth exceeding 35 GHz in the W-band was used. The second stage is frequency conversion, as shown in Fig.S3. It begins with the on-chip modulator which upconverts the wireless signal to the optical domain, and ends with the balanced photodetectors (BPD) which recover the baseband signal. Based on previous theoretical studies[2,3], we derived the expression for conversion loss in the proposed on-chip photonic architecture as:

$$G_2 = \left(\frac{Z_l R \pi}{2V_\pi}\right)^2 \cdot P_{\text{in}} \cdot P_{\text{Lo}} \tag{3}$$

where Z_l represent the input and output load of modulator and BPD of 50 Ω. R represents the BPD's responsivity of around 0.25 A/W. Since we used the phase modulator for wireless-photonic conversion during the experiment, its V_π is twice that of a MZ modulator (2.8 V), while the phase modulator avoids the intrinsic 3 dB optical loss. P_{in} represents the optical power injected into the phase modulator of around 5 dBm, excluding the overall link loss. P_{Lo} represents the power of the sideband generated by the on-chip OEO, which is approximately 5 dBm in the experiment. Using the above parameters, the conversion loss is calculated to be 39.1 dB. The expression of NF can

be written as[4]:

$$F_2 \equiv \frac{N_{\text{out}}}{G_2 k_b T} \tag{4}$$

where k_b is the Boltzmann constant, T is the standard temperature (290 K), and N_{out} is the output noise power. In our coherent system, thermal and shot noise are the dominant contributors. Based on G_2 of the microwave photonic link, the resulting NF is calculated to be 41.1 dB. Therefore, the overall link noise figure is approximately 8.21 dB in our system. We further evaluated the effect of different V_π and photodetector responsivity R on NF, as shown in Fig.S4. The results indicate that even with a relatively high V_π of 9 V, the noise figure remains acceptable at 10.52 dB when the responsivity R of 0.3 A/W. Meanwhile, we experimentally measured the gain and noise figure of the entire photonic-assisted link and calculated the overall link noise figure using the Friis equation, yielding a value of approximately 12.63 dB. The degradation is likely due to the optical receiver introducing additional optical loss via power splitting for dual-polarization operation, but not used in our work. This equivalently reduces the responsivity R of the BPD.

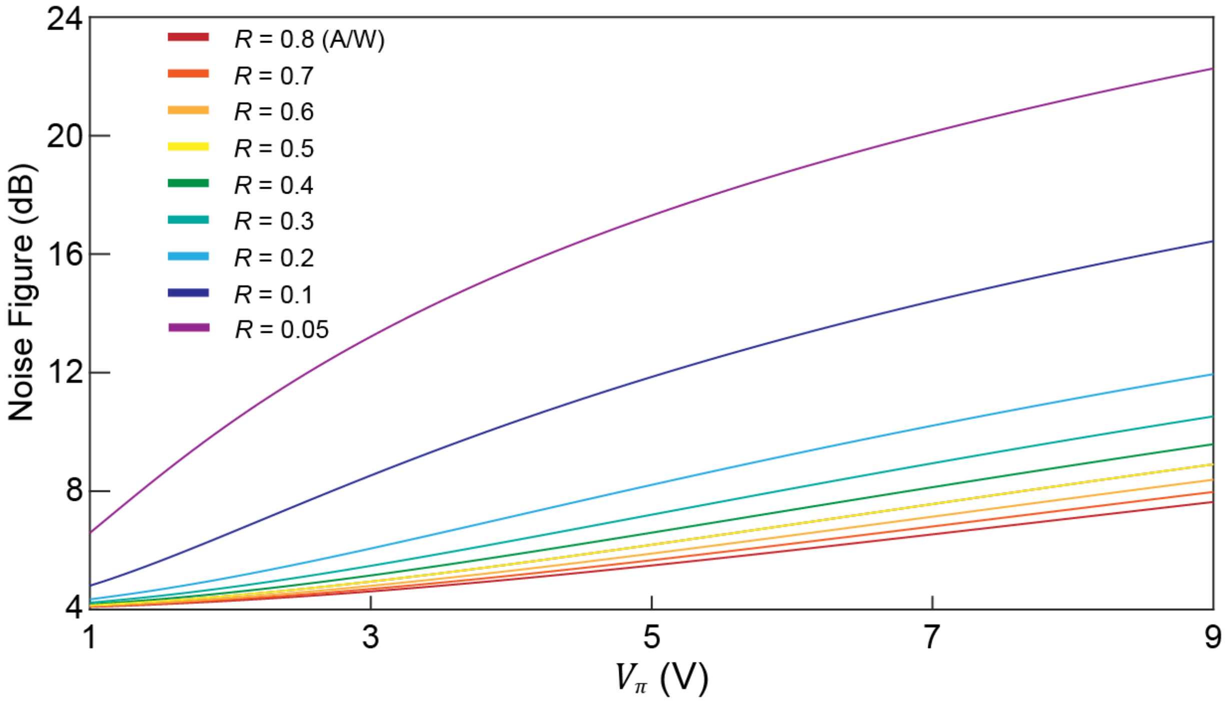

Fig. S4. Calculated noise figure as a function of V_π and R

The key advantage of the proposed scheme lies in its ultrabroadband signal processing capability. The on-chip photonic architecture separates the conventional electronic mixer functionality across three distinct components: the modulator (RF port), the BPD (IF port), and the passive

optical hybrid (LO port), as illustrated in Fig.S3. We incorporated the frequency responses of the modulator, amplifier in W band, and BPD to estimate the NF as a function of both input RF bandwidth and IF bandwidth, as shown in Fig.S5. Our scheme can maintain a flat NF over 35 GHz RF and 50 GHz IF bandwidths, and the RF range can be further extended to 70 GHz when using a commercially available higher-bandwidth amplifiers, with over 30 dB gain from 0.05–70 GHz and an in-band noise figure ranging from 4 to 6 dB. In contrast, electronic implementations exhibit narrower bandwidths (Table 1), mainly due to rising mixer loss beyond the operating range. For the LO port, the passive optical hybrid usually supports broadband operation in the optical domain (tens of nanometers), so unlike electronic mixers, it does not require dedicated broadband design strategies. Our multi-band wireless experiments have confirmed communication with LO tuning from 5 to 100 GHz, indicating that the effective noise figure bandwidth for LO frequency exceeds that range, which significantly outperforms electronic counterparts.

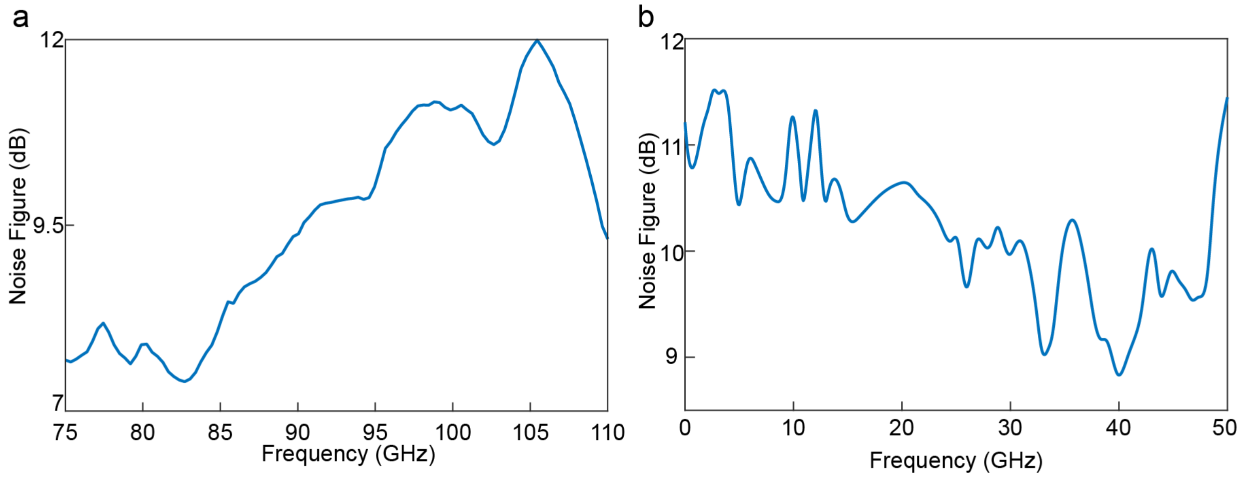

Fig. S5. Noise figure as a function of frequency of RF port (**a**) and IF port (**b**)

Supplementary note IV: Detailed characterization of on-chip OEO

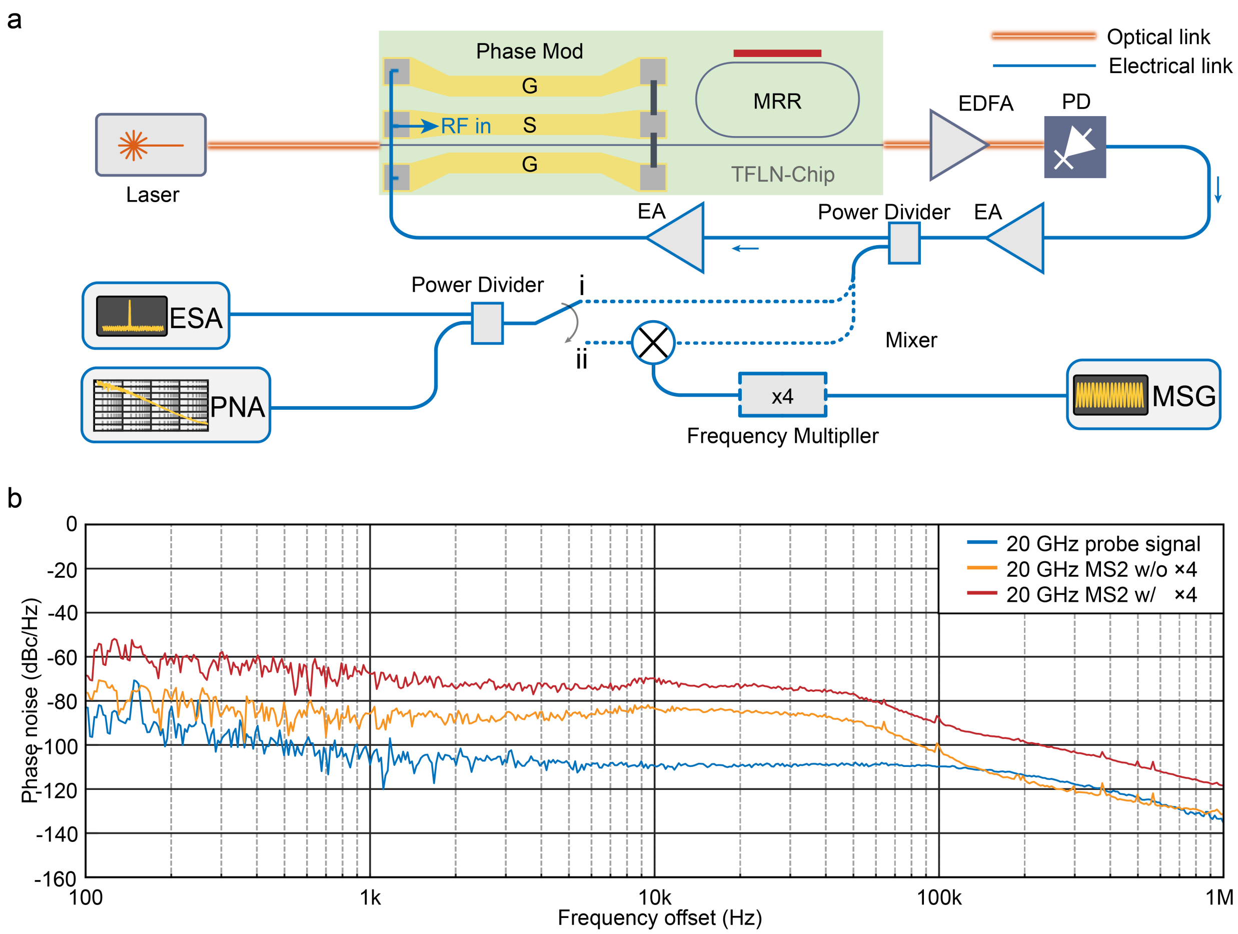

Fig. S6. **a,** Setup for OEO oscillation frequency measurement and microwave signal phase noise measurement in low-frequency(5 GHz and 20 GHz, when the switch in the link diagram is set to position i.) and high frequency (90 GHz, when the switch in the link diagram is set to position ii). **b,** Microwave source phase noise with and without the frequency-multiplying and mixing link, used to confirm the phase noise degradation introduced by x4 frequency multiplier and down-mixing detection. ESA: electronic spectrum analyzer; PNA: phase noise analyzer; MSG: microwave signal generator.

The experimental setup for getting OEO oscillation frequency and generated microwave signal phase noise is shown in Fig.S6a. To measure the phase noise of the high-frequency signals as 90 GHz, we employ a 20 GHz probe signal from a commercial microwave source (Keysight E8257D) with phase noise of -110 dBc/Hz at 10 kHz frequency offset(blue curve in Fig.S6b, noted as MS1). This probe signal is quadrupled and mixed with the OEO signal to down-convert high-frequency signals to 10 GHz. The test results demonstrate that the RF signals at 5 GHz, 20 GHz, and 90 GHz consistently exhibit a phase noise level of -85 dBc/Hz at 10 kHz frequency offset, confirming phase noise consistency across both low and high frequencies. Subsequently, we add a 2 km fiber

to the oscillation loop. Measurements indicate that the 5 GHz and 20 GHz signals maintain the same phase noise of -110 dBc/Hz at 10 kHz. However, the phase noise of the 90 GHz signal is measured at -100 dBc/Hz at 10 kHz, as shown in Fig.2h of the main text. This degradation is induced by the x4 frequency multiplier and down-mixing detection scheme, where the probe signal incurs an additional 10 dB phase noise, masking the inherent phase noise of the on-chip generated signal. To further verify this, a second microwave source (Anritsu, MG3695A) with a phase noise of about -80 dBc/Hz at 10 kHz frequency offset at 20 GHz (orange curve in Fig.S6b, noted as MS2) is also quadrupled and mixed with the quadrupled probe signal generated by Keysight E8257D. The phase noise of the beat note is -70 dBc/Hz at 10 kHz frequency offset(red curve in Fig.S6b), showing an approximately 10 dB degradation. This indicates that the frequency multiplication and mixing operations indeed introduce around 10 dBc/Hz at 10 kHz frequency offset degradation in phase noise.

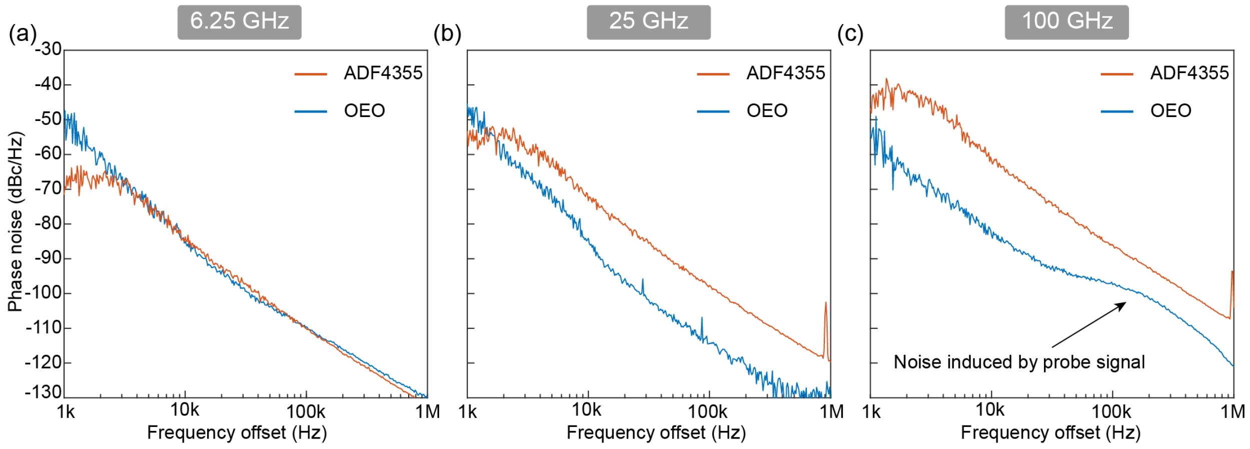

Fig. S7. Phase noise measure at 6.25 GHz (**a**), 25 GHz (**b**) and 100 GHz (**c**)

To evaluate the frequency stability of the free-running oscillation, we conduct a one-hour test at an oscillation frequency of approximately 20 GHz. Spectral data is collected at one-minute intervals using an electrical spectrum analyzer (Keysight 9010B) with a scan width of 100 kHz and a video bandwidth of 910 Hz. Setup for frequency stability experiment is the same as the low-frequency section of the OEO spectrum testing link(Fig.S6a). Each dataset undergoes Lorentzian fitting, from which we extract the central frequency.

In comparison to conventional electronic schemes, the OEO approach maintains consistent phase noise as the oscillation frequency increases, whereas in frequency multiplier chains, phase noise degrades proportionally with the multiplication factor. The relationship between the multiplication

factor N and noise accumulation can be expressed as:

$$\Delta\mathcal{L} = 20 \cdot \log_{10}(N) \tag{5}$$

where $\Delta\mathcal{L}$ represents the additional phase noise. For quantitative comparison, we employ a commercially available frequency-synthesizer chip followed by a multiplier as the benchmark reference. The ADF4355 frequency synthesizer from Analog Devices (ADI), which has been extensively deployed in current wireless systems for low-noise LO generation, integrates VCO and PLL locking circuitry to ensure low phase noise, with a maximum output up to 6.8 GHz. In the experiment, the synthesizer was set to 6.25 GHz and then up-converted to 25 GHz and 100 GHz via two cascaded $\times 4$ frequency multipliers. The resulting phase-noise spectra (orange traces, Fig.S7a-c) show that the noise at a 10 kHz offset degrades from -85 dBc/Hz to -73 dBc/Hz and -62 dBc/Hz, respectively. By contrast, the on-chip OEO approach yields phase noise that remains virtually unchanged with frequency. Across all three frequencies, the blue traces in Fig.S7 reveal -85 dBc/Hz at 10 kHz offset even at 100 GHz, clearly outperforming the multiplier-based electronic approach.

Supplementary note V: Demonstration and analysis of further broadband compatibility with electronic devices

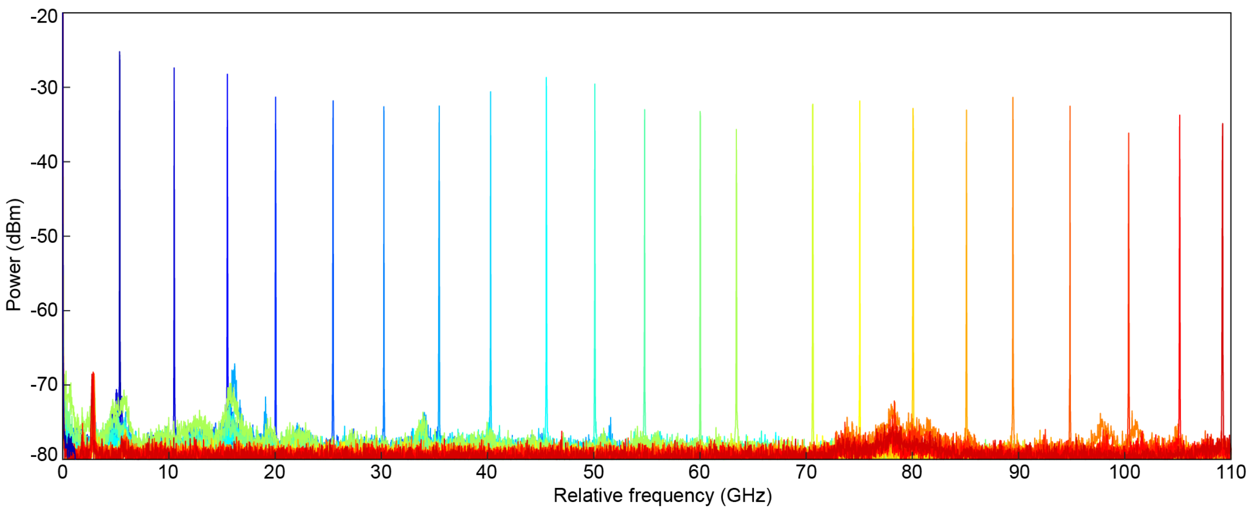

Fig. S8. Optical spectrum of the OEO output sideband signal, measured by an optical spectrum analyzer

Our work, by leveraging on-chip components and introducing a system-level architecture, demonstrates the potential of the TFLN platform for future 6G wireless communications. This system-level architecture is enabled by integration technologies that combine multiple photonic devices within a single TFLN chip. At the device level, as briefly summarized in Supplementary I, we have achieved key performance metrics in modulation bandwidth, optical insertion loss, and Q factor, and, more importantly, developed scalable fabrication processes that support the reliable integration of multiple TFLN devices into a single chip-scale system. This combination of performance and scalability enabled the first demonstration of an on-chip optoelectronic oscillator (OEO) with an operating bandwidth exceeding 110 GHz and continuous tunability across the entire range.

To further demonstrate the wideband tunability of the OEO scheme, we implemented custom-designed broadband amplifiers, so the OEO experiment now generates a widely tunable LO signal using a single set of broadband electronic amplifiers. The results are presented in Fig.S8. We fabricated a new TFLN chip, in which the on-chip microring features an FSR exceeding 200 GHz for PM-IM mode selection. To record data across the wide frequency span, we used an optical spectrum analyzer to capture the sidebands and verify the full band signals. This time, without the need to replace amplifiers for different frequency ranges, we can generate LO signals from low frequency to 110 GHz simply by adjusting the microring resonator.

The bandwidth and agility demonstrated in our work were previously unattainable in both

fiber-based and integrated platforms. The advanced on-chip OEO source plays a crucial role as an enabler for both transmission and reception in wireless communication systems. By further integrating key functionalities in end-to-end link, as illustrated in Fig.1c of the manuscript, our work enables a fundamental transformation in hardware architecture for wireless communication systems in order to meet the anticipated demands of 6G, including higher data rates, greater adaptability in complex application scenarios, and enhanced flexibility. It delivers two critical attributes for wireless transceivers: (1) consistent ultra-wideband performance and (2) flexible full-spectrum reconfigurability, both of which are highly difficult to achieve with current electronic solutions.

Moreover, it is important to analyze the broadband compatibility of the antenna technologies for the proposed system. Recent advances in semiconductor and meta-surface technologies have reinforced its feasibility for antennas.. One approach is to realize electronically tunable antennas with the assistance of metasurface[5], in which materials such as ferrites, liquid crystal or graphene can flexibly tune the resonant frequency[6–10]. Another approach employs MEMS/NEMS technologies to physically adjust the size of radiating elements, thereby altering the resonant frequency[11–13]. Therefore, these advanced materials and technologies hold promise for achieving tunability across sub-6 GHz, mmWave, and THz frequency bands. Moreover, antenna-coupled modulators based on the TFLN platform can also achieve broadband receiving[14].

Meanwhile, we also list several approaches to realize integration between the proposed system and other electronic components. Regarding the photonic-electronic integration question, many practical solutions have been recently established and adopted in the integrated photonics community toward photonic-electronic co-packaging and integration. Such efforts include heterogeneous integration, 3D integration[15,16], and advanced 2.5D packaging techniques, including flip-chip bonding with high bandwidth design[17], to enable seamless integration between electronic amplifier chips and modulator chips. These strategies aim to meet the growing demands for higher bandwidth and faster data rates. A particularly promising candidate for achieving 2.5D co-packaging of TFLN-based-photonic/electronic chips, as well as other components like antennas, is glass interposers[18,19]. In terms of electrical performance, glass offers inherently low microwave loss, and to the best of our knowledge, transmission line designs on glass have already demonstrated operation beyond 100 GHz, supporting the wideband requirements of our system. In addition, broadband antennas have also been demonstrated on glass interposers, directly enabling 6G-related wireless applications[20]. Meanwhile, several demonstrations have already validated the capability of glass interposers to support optical signal propagation[21,22]. Therefore, it offers strong potential for enabling

inter-chip optical interconnects on the same interposer platform, such as between laser, modulator and BPD chips.

Also, we discussed the challenges for further extending the bandwidth of the optoelectronic devices towards THz bandwidth. The key to further improve the bandwidth of on-chip modulators is to minimize the microwave loss in the transmission line while maintaining velocity and impedance matching. Our preliminary results have shown that TFLN modulators can effectively operate at up to 500 GHz, deep into the THz spectral range, with a measured RF half-wave voltage of 8 V at 500 GHz[23]. For high-bandwidth photodetectors, the key challenge is to minimize the intrinsic carrier transit time. UTC-PDs address this by employing electron-only transport, which eliminates the slow hole transit time and enables ultrahigh-bandwidth operation exceeding hundreds of gigahertz[24,25].

Finally, we envision two practical deployment scenarios, where our system could be deployed immediately. 1) 6G wireless infrastructure: In the near future, 6G networks will require diverse wireless devices for various applications. Our system can serve as a photonic core, plugged for frequency-agnostic signal conversion to the target bands, with other peripheral components adapted as needed. This design would significantly reduce hardware heterogeneity across devices within the network. The costs of separate customization, maintenance, and upgrading of each frequency band would be significantly reduced. It can also support higher data rates thanks to its broadband capability. Moreover, this allows the base station to interface more seamlessly with the optical fiber network, eliminating the need for additional E/O conversion modules; 2) Integration with phased arrays: Our scheme can also be integrated into phased array systems, where multiple antennas share a common RF chain. This implies that a single photonic mixer could support frequency mixing across the entire antenna array, significantly simplifying the system architecture. Furthermore, since dual-band and multiband phased arrays have already been well demonstrated[26–28], we may take a step further to envision an architecture in which antenna elements operating at different frequencies are integrated within a single array and uniformly upconverted into the optical domain for unified processing.

Supplementary note VI: Experimental details for wireless communication quality optimization

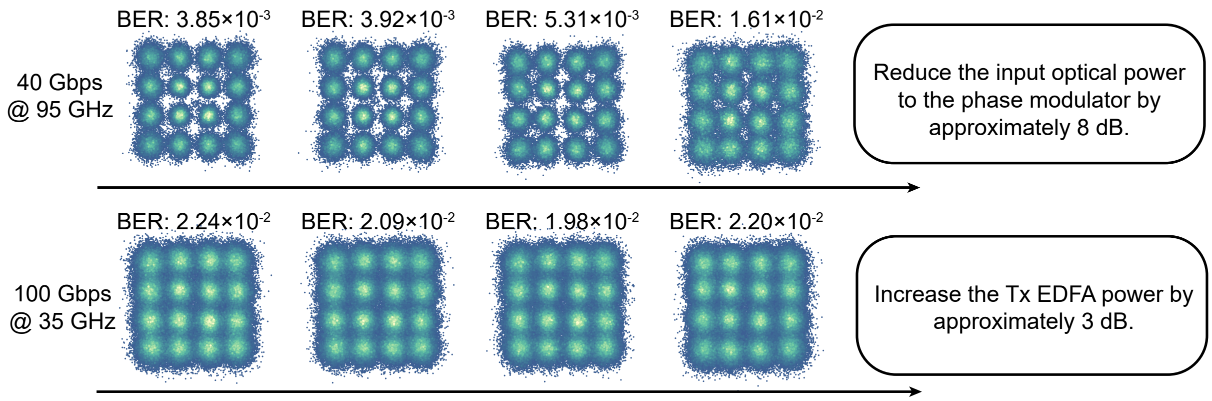

Fig. S9. Optimizing BER by adjusting component.

Optimizing the signal-to-noise ratio (SNR) is a key strategy for enhancing communication quality in the proposed systems. First of all, the modulator which is directly connected to the antenna, is responsible for converting wireless signals into the optical domain. This conversion efficiency directly determines the SNR of the communication. During the experiment, we found that the optical insertion loss of this on-chip modulator should be minimized to mitigate the degradation of the SNR. Fortunately, as stated in the main text, implementing the Pockels effect introduces minimal loss to the optical power in the waveguide. Here, the optical insertion loss of the fabricated TFLN-based modulator can be maintained below 2 dB, which, compared to other platforms, further enhances communication quality and is one of the reasons for us to realize high transmission rates. To further validate it, we gradually reduced the power of the injected optical power, then tested and recorded the BER of the wireless communication. The results indicate that a total additional loss of approximately 8 dB would degrade the communication error rate from 3.85×10^{-3} to 1.61×10^{-2}.

Moreover, optimizing the gain at each node in the wireless communication link is also crucial. For instance, at Tx end, the EDFA positioned before the PD, which can adjust the transmitted signal's intensity, must operate at an optimal gain level to achieve the highest communication quality. Lower gain levels can lead to insufficient power in the wireless signal, whereas higher gain levels might result in saturation of the electrical amplifier connected behind the antenna at Rx end. Consequently, the gain must be carefully balanced to achieve the optimal SNR. Fig.S9 illustrates this strategy for optimizing the SNR. By gradually fine-tuning the gain of the EDFA by approximately 3 dB, the BER initially increased from 2.24×10^{-2} to 1.98×10^{-2}, but then

decreased to 2.2×10^{-2}, due to approaching electrical amplifier's output saturation. We conducted multiple sets of tests, and all of them showed similar trends.

Supplementary note VII: Performance characterization of channel hopping speed

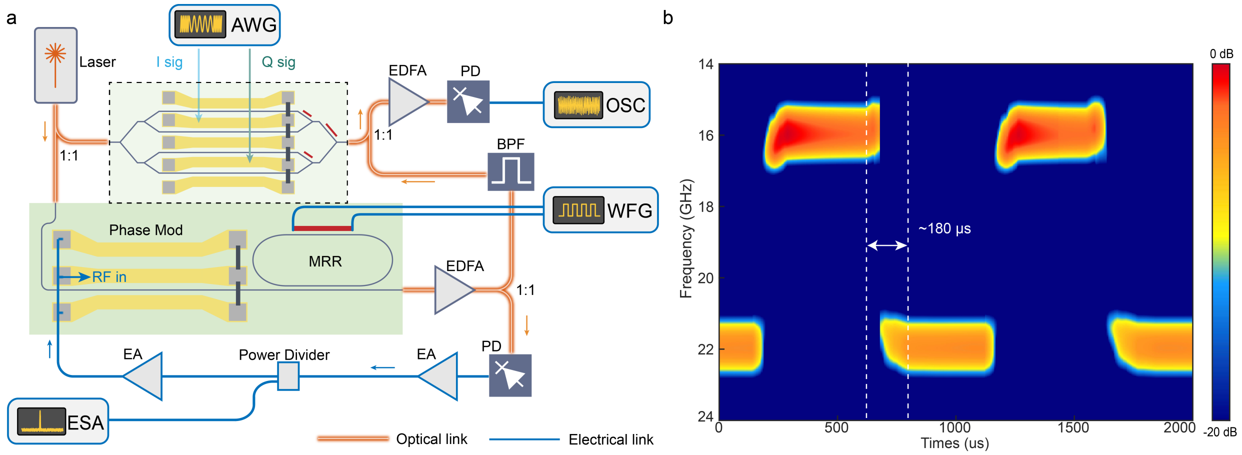

Fig. S10. **a**, Setup for two channels switching experiment. **b**, Time-frequency analysis results of the frequency-hopping signal. WFG: waveform generator; OSC: oscilloscope; AWG: any waveform generator.

Here we characterize the switching speed of center frequency tuning in wireless signals. By controlling the voltage applied on the microheater of the MRR, the center frequency of the wireless signal can be shifted by the thermo-optics effect. The measurement setup is shown in Fig.S10a. During the experiment, we achieve frequency channel hopping by applying a square wave signal from the waveform generator (WFG) to the microheater. The frequency spacing between channels is determined by the amplitude of the square wave signal. By applying a 1 kHz square wave signal with an amplitude of 2.8 V, the wireless communication channel can be switched from 16 GHz to 22 GHz. Fig.S10b shows the time-frequency analysis of a wireless signal, with a digital filter to filter out the intermediate transiting frequency state between two channels for clear observation. The measured channel switching time interval is approximately 180 μs, which is primarily limited by the response speed of the thermo-optics effect and can be further improved by using the Pockels effect with femtosecond timescale response.

Supplementary note VIII: Analysis of the impact non-ideal frequency response has on communication performance

We first measure the ripples and dips present in our wireless channel. We used a vector network analyzer (VNA) to test the gain curves of the low-noise amplifiers (LNAs) (75–110 GHz) at both the transmitter and receiver. The sampling interval is 500 MHz, and the VNA's output power was maintained within the linear amplification range of the LNAs. The gain curves of the two LNAs exhibited nearly identical characteristics. As shown in the gain curves presented in Fig.4b of the main text, the gain is relatively flat in the 90–110 GHz frequency range, while a steep increase occurs between 75–90 GHz. Additionally, sharp gain peaks are observed around 83 GHz and 95 GHz, with dips occurring around 78 GHz and 90 GHz. As a result, this uneven gain profile introduces significant challenges for maintaining high communication quality, particularly for advanced modulation formats such as 16-QAM, which are sensitive to amplitude and phase distortions.

Supplementary note IX: Experimental details of the active interference avoidance

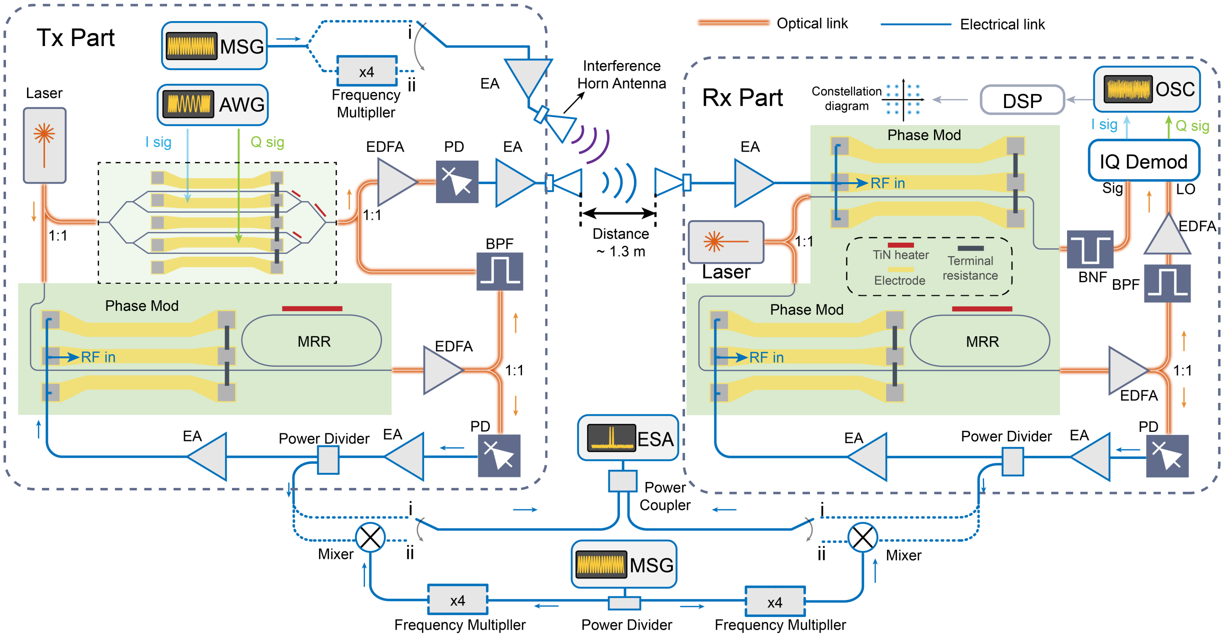

Fig. S11. Setup for active interference avoidance in low-frequency(∼25 GHz, when the switch in the link diagram is set to position i.) and high frequency(∼96.55 GHz, when the switch in the link diagram is set to position ii). MSG: microwave signal generator; AWG: any waveform generator; ESA: electronic spectrum analyzer; OSC: oscilloscope.

The complete experimental setups of the active interference avoidance in low (25 GHz) and high frequency (96.55 GHz) are illustrated in Fig.S11. We use the microwave source generation (MSG, Keysight E8257D) to generate the single-tone microwave to serve as the interference. For generating high-frequency signals beyond the MSG's available range, we employed a $\times 4$ frequency multiplier to up-convert the output frequency into the high-frequency band. Then we introduce additional antennas to emit the interference signals, which will overlap with the target signal during wireless propagation in space and be jointly received by the receiving antenna. In Fig.S11, when the switch in the link diagram is set to position i, the microwave source output signals have amplitudes and frequencies as follows: 6 dBm at 25 GHz, 0 dBm at 25 GHz, 6 dBm at 23 GHz, and 6 dBm at 21 GHz. When the switch in the link diagram is set to position ii, we displays signals with the following specifications: 8 dBm at 24.125 GHz, 9 dBm at 24.375 GHz, 9 dBm at 24.625 GHz, and 10 dBm at 24.875 GHz. Note that this does not represent the actual transmitted signal power, as the frequency multiplier exhibits slight gain variations at different frequency points. The specific power of the interference signals and their power ratio to the target signal can be clearly observed and compared in Fig.4.f and 4.g of the main text. As for the Rx end, a digital low-pass filter

(LPF) is selected to eliminate the out-of-band noise signal during signal recovery. Typically, this low-pass filter is positioned 1 GHz beyond the baseband signal's bandwidth. For a 10 GBaud QPSK signal, the passband can be set to 0-6 GHz. Experiments demonstrate that the interference patterns affecting the signal are fundamentally similar for both low-frequency and high-frequency carriers. When interference occurs outside the signal's spectrum range and is filtered out by a low-pass filter, its impact on signal transmission quality is minimal.

Supplementary note X:Low-power-consumption demonstration of the wireless communication

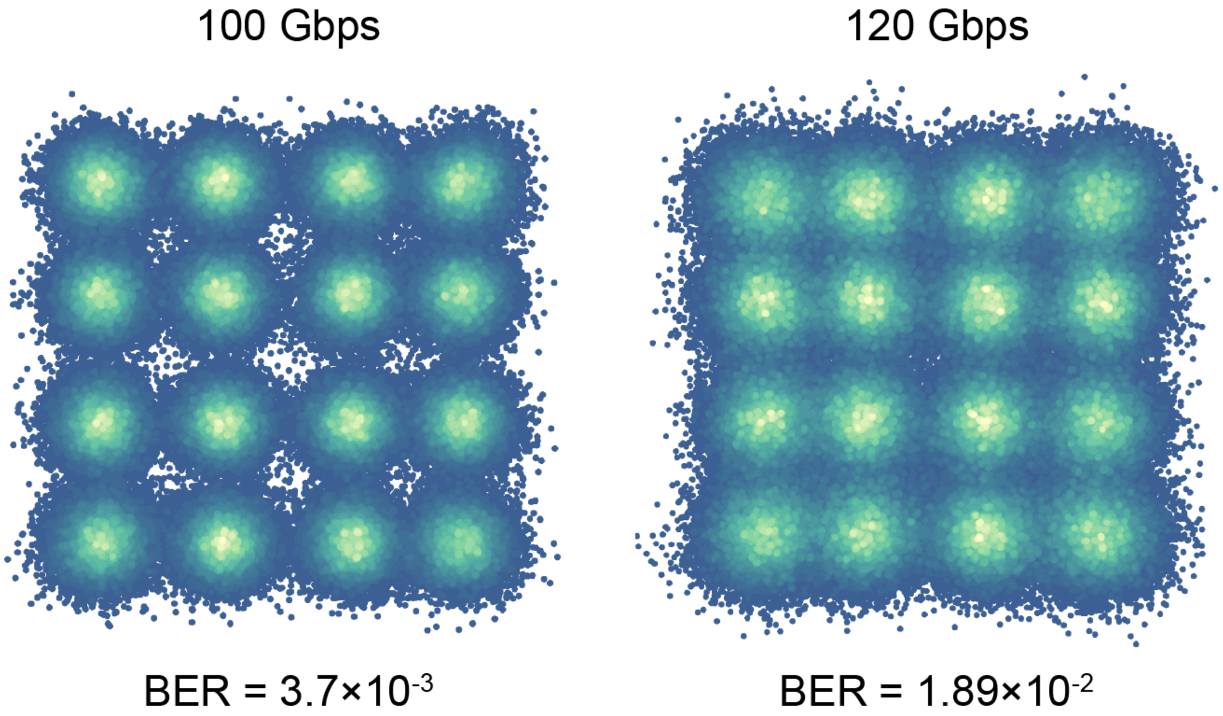

Fig. S12. Measured constellation diagrams in low consumption demonstration

To demonstrate the feasibility of lower power operation, we optimized the butt-coupler insertion loss and packaging losses of the TFLN chip (2–3 dB per facet), allowing us to eliminate the need for power-hungry EDFAs. We then present a power consumption breakdown of the wireless communication setup, along with the demodulation results. Both the transmitter and receiver require a laser, each consuming approximately 0.5 W. The laser output (∼14 dBm) is split by a 3 dB coupler into two paths: one for the OEO and the other for signal processing. For the OEO path, the EDFA before the PD is not needed. The power consumption of this path includes two cascaded electrical amplifiers (0.28 W) and the photodetector (0.0012 W).For the signal processing path at the transmitter side, the two electronic drivers for the IQ modulator consume 0.6 W. A semiconductor optical amplifier (SOA) chip provides minor optical gain compensation, consuming 0.8 W. The amplified optical signal is then sent to the photodetector (0.003 W) and further amplified by an electronic amplifier (0.21 W) before being transmitted by the Tx antenna.At the receiver side, the signal is first amplified by a low-noise amplifier (0.24 W) before being sent into the receiver modulator for wireless-to-photonic conversion. The LO optical signal generated by the

OEO section is further amplified by a second SOA (0.8 W) and then fed into the optical coherent receiver, where the TIAs and balanced photodetectors consume approximately 0.66 W. The overall link consumption is 4.8754 W.

Fig.S12 shows the low-power wireless communication results, which also demonstrated a high-speed data rate of 100 Gbps and 120 Gbps at 35 GHz, with the error bit rate (BER) of 3.7×10^{-3} and 1.89×10^{-2}, respectively. This result, even better than the results in Fig.3 in the main part, highlights that minimizing optical loss is critical to significantly enhancing performance and reducing power consumption in photonic-assisted wireless communication.

---

[1] Kharel, P., Reimer, C., Luke, K., He, L. & Zhang, M. Breaking voltage-bandwidth limits in integrated lithium niobate modulators using micro-structured electrodes: erratum. *Optica* **8**, 1218–1218 (2021).

[2] Clark, T. R., O'Connor, S. R. & Dennis, M. L. A phase-modulation i/q-demodulation microwave-to-digital photonic link. *IEEE Transactions on Microwave Theory and Techniques* **58**, 3039–3058 (2010).

[3] Noe, R. Consistent optical and electrical noise figure. *Journal of Lightwave Technology* **41**, 137–148 (2023).

[4] Urick, V. J., Williams, K. J. & McKinney, J. D. *Fundamentals of microwave photonics* (John Wiley & Sons, 2015).

[5] Li, A., Singh, S. & Sievenpiper, D. Metasurfaces and their applications. *Nanophotonics* **7**, 989–1011 (2018).

[6] Taghvaee, H. *et al.* Tunable graphene-based metasurfaces for multi-wideband 6g communications. In *2022 Sixteenth International Congress on Artificial Materials for Novel Wave Phenomena (Metamaterials)*, 434–436 (IEEE, 2022).

[7] Lee, S. H. *et al.* Switching terahertz waves with gate-controlled active graphene metamaterials. *Nature materials* **11**, 936–941 (2012).

[8] Sharma, M., Pathak, S. & Sharm, M. Fmr measurements of magnetic nanostructures. *Ferromagnetic Resonance-Theory and Applications* 93–110 (2013).

[9] Yang, H., Yu, T., Wang, Q. & Lei, M. Wave manipulation with magnetically tunable metasurfaces. *Scientific Reports* **7**, 5441 (2017).

[10] Guirado, R., Perez-Palomino, G., Ferreras, M., Carrasco, E. & Caño-García, M. Dynamic modeling of liquid crystal-based metasurfaces and its application to reducing reconfigurability times. *IEEE Transactions on Antennas and Propagation* **70**, 11847–11857 (2022).

[11] Akyildiz, I. F., Kak, A. & Nie, S. 6g and beyond: The future of wireless communications systems. *IEEE Access* **8**, 133995–134030 (2020).

[12] Aboagye, S. *et al.* Multi-band wireless communication networks: Fundamentals, challenges, and re-

source allocation. *IEEE Transactions on Communications* (2024).

[13] Akyildiz, I. F., Jornet, J. M. & Nie, S. A new cubesat design with reconfigurable multi-band radios for dynamic spectrum satellite communication networks. *Ad Hoc Networks* **86**, 166–178 (2019).

[14] Gaier, A. *et al.* Antenna-coupled integrated millimeterwave modulators and resonant electro-optic frequency combs. *arXiv preprint arXiv:2505.04585* (2025).

[15] Atabaki, A. H. *et al.* Integrating photonics with silicon nanoelectronics for the next generation of systems on a chip. *Nature* **556**, 349–354 (2018).

[16] Daudlin, S. *et al.* Three-dimensional photonic integration for ultra-low-energy, high-bandwidth interchip data links. *Nature Photonics* 1–8 (2025).

[17] Li, K. *et al.* An integrated cmos–silicon photonics transmitter with a 112 gigabaud transmission and picojoule per bit energy efficiency. *Nature Electronics* **6**, 910–921 (2023).

[18] Sawyer, B. M. *et al.* Design and demonstration of a 2.5-d glass interposer bga package for high bandwidth and low cost. *IEEE Transactions on Components, Packaging and Manufacturing Technology* **7**, 552–562 (2017).

[19] Usman, A. *et al.* Interposer technologies for high-performance applications. *IEEE Transactions on Components, Packaging and Manufacturing Technology* **7**, 819–828 (2017).

[20] Huang, K.-Q. & Swaminathan, M. Antenna array on glass interposer for 6g wireless communications. *IEEE Transactions on Components, Packaging and Manufacturing Technology* **13**, 211–218 (2023).

[21] Brusberg, L. *et al.* Glass interposer for high-density photonic packaging. In *2022 Optical Fiber Communications Conference and Exhibition (OFC)*, 1–3 (IEEE, 2022).

[22] Brusberg, L. *et al.* Glass platform for co-packaged optics. *IEEE Journal of Selected Topics in Quantum Electronics* **29**, 1–10 (2023).

[23] Zhang, Y. *et al.* Monolithic lithium niobate photonic chip for efficient terahertz-optic modulation and terahertz generation. *arXiv preprint arXiv:2406.19620* (2024).

[24] Lischke, S. *et al.* Ultra-fast germanium photodiode with 3-db bandwidth of 265 ghz. *Nature Photonics* **15**, 925–931 (2021).

[25] Shi, Y. *et al.* Avalanche photodiode with ultrahigh gain–bandwidth product of 1,033 ghz. *Nature Photonics* **18**, 610–616 (2024).

[26] Wang, S. & Rebeiz, G. M. Dual-band 28-and 39-ghz phased arrays for multistandard 5g applications. *IEEE Transactions on Microwave Theory and Techniques* **71**, 339–349 (2022).

[27] Valavan, S. E., Tran, D., Yarovoy, A. G. & Roederer, A. G. Dual-band wide-angle scanning planar phased array in x/ku-bands. *IEEE transactions on antennas and propagation* **62**, 2514–2521 (2014).

[28] Chekole, B. Z., Salau, A. O. & Kassahun, H. E. Multiband millimeter wave phased array antenna design for 5g communication. In *2022 International Conference on Innovation and Intelligence for Informatics, Computing, and Technologies (3ICT)*, 106–111 (IEEE, 2022).
